# Assessing complex executive functions with computerized tests: is that toast burning?

**DOI:** 10.3389/fnbeh.2014.00362

**Published:** 2014-10-22

**Authors:** Brian E. McGuire

**Affiliations:** School of Psychology, National University of IrelandGalway, Ireland

**Keywords:** brain injury, chronic, executive function, psychometric test, compuetrized test, neuropsychological assessment

Can a computer simulate the smell of burning toast? The paper by Tanguay et al. (2014) examined the utility of a computer simulated cooking task for people with an acquired brain injury. The paper highlights an important challenge in clinical neuropsychology—that of developing methods for testing everyday functioning without having to be in everyday situations.

But why is this important? There are already a great many questionnaires used to assess functional capacity after brain injury. However, it is now well recognized that people with brain injury may have impaired self-awareness and thus may not be able to provide an accurate self-assessment of their abilities (McBrinn et al., 2008; Caldwell et al., 2014). The reliability of third-party report from caregivers has also been questioned (Barker et al., 2011; McGuire et al., 2014). There is therefore a significant benefit in conducting assessment of real-life performance of functional tasks. Clinically, it is preferable to assess functional performance in the context within which the skills are to be applied—this is the best way to know how a person will perform in a given task or situation. Tests such as the Multiple Errands Test which is in essence a shopping task (Shallice and Burgess, 1991) and the Executive Function Performance Test (simple cooking, telephone use, medication management, and bill payment) (Baum et al., 2008), apply this principle capably, by using the natural environment as the “laboratory” but while also applying a degree of scientific rigor through the use of a standardized testing protocol.

However, the conduct of tests in their naturalistic environment poses a number of challenges for both clinicians and researchers. For example, there are very practical considerations such as the availability of a suitable environment in which to conduct testing. Evaluating the ability to negotiate the multiple aisles of a large supermarket may not be easy for those in rural areas where there may not be a large market within easy commuting distance. Being able to regulate a gas-operated stove, which is qualitatively different to cooking with an electric stove, will depend on the availability of gas in the area in which testing is being done. Assessing the ability to catch the right bus and alight at the correct stop poses logistical demands on the assessor. There are also possibly additional health and safety challenges associated with testing people with impaired abilities in the naturalistic environment. Arguably it is also more difficult to standardize the evaluation process in a situation where the very nature of the environment is that is not standardized: buses run late, the products change in shop aisles, each cooker is a little bit different. These challenges highlight a tension between the benefits of ecologically valid testing and the practical difficulties this type of testing entails.

In this context, a small number of computerized tests have been developed to simulate functional tasks of everyday life such as working in an office environment (Lamberts et al., 2010) or a virtual version of the multiple errands task (Rand et al., 2009). The paper by Tanguay et al. is an example of the growing interest in harnessing the capabilities of computer technology to evaluate functional abilities. It is easy to understand the appeal of this approach. For example, standardized testing is much easier to achieve—the assessor determines the parameters to be tested; automated recording is possible, such as reaction time or time taken to achieve a task; the need to have an “actual” testing environment is removed; the testing can be done without any need for special planning so logistical demands are negated; environmental hazards are removed and it may also reduce the demand on therapist time.

However, the cost of developing these technologies is significant and there may still be a significant gap between the technology used in computer simulations and the scientific requirements associated with psychometric testing. It is also the case that computer simulations cannot fully replicate the uncertainties of everyday life. In the paper by Tanguay et al. the test demands focused on timing of food preparation and setting a table. However, what happens in a real kitchen is multisensorial—one will hear the microwave bing and the kettle whistling, smell the toast burning and visualize the eggs to see if they are cooking evenly. It is understandable that there may be concern that the ecological validity of a test will be compromised if the test is not conducted in the relevant naturalistic environment. However, the rapid evolution of interactive computing such as that used in serious gaming and virtual reality applications points to the potential for exceptionally “life-like” testing environments, including 4-D simulations which can include a variety of sensory stimuli such as vibration, odors, and tactile components. Ultimately, the value of computerized simulations will be determined by the extent to which they can predict everyday functional performance in the real world. The study by Tanguay has made a useful contribution to this field but also highlights the ongoing challenge to maximize the potential of computer simulations within the complex world of clinical practice.

## Conflict of interest statement

The author declares that the research was conducted in the absence of any commercial or financial relationships that could be construed as a potential conflict of interest.
